# Prevalence of work-related musculoskeletal disorders and associated factors among University of Zimbabwe Faculty of Medicine and Health Sciences non-academic workers: a cross-sectional study

**DOI:** 10.1186/s12891-023-06900-1

**Published:** 2023-10-06

**Authors:** Letwin Nomalungelo Tembo, Jacquiline Paidamoyo Munyikwa, Chipo Musoro, Grace Majonga, Edwin Mavindidze

**Affiliations:** Occupational Therapy Program, Rehabilitation Sciences Unit, University of Zimbabwe, Faculty of Medicine and Health Sciences, Rehabilitation Sciences Unit, Avondale, P.O. Box A178, Harare, Zimbabwe

**Keywords:** Work-related musculoskeletal disorders, Prevalence, University

## Abstract

**Background:**

Work-related musculoskeletal disorders most commonly contribute to years lived with disability among workers. Heavy physical work, static work posture, awkward posture, force exertion, lifting and repetitive movements increase the risk of developing work-related musculoskeletal disorders.

**Objectives:**

The aim of the study was to determine the prevalence of work-related musculoskeletal disorders and associated factors among non-academic workers at the University of Zimbabwe Faculty of Medicine and Health Sciences. Non-academic workers included security personnel, catering staff, drivers, library staff, clerical staff, technicians and janitorial staff.

**Methods and materials:**

The study used a cross-sectional analytical design. One hundred and eight non-academic workers at the University of Zimbabwe Faculty of Medicine and Health Sciences were sampled through proportional stratified sampling in January 2021. An adapted Nordic Musculoskeletal Questionnaire capturing socio-demographics and ergonomic risk factors was used to gather data. SPSS v24 was used for data analysis including frequencies, tests of association and multivariate logistic regression.

**Results:**

One hundred non-academic workers from the University of Zimbabwe Faculty of Medicine and Health Sciences responded. The 3-month and 12-month prevalences of work-related musculoskeletal disorders were highest in the lower back (*n* = 72, 72% and *n* = 75, 75%) and wrists/hands (*n* = 60, 60% and *n* = 69, 69%) respectively. Sociodemographic factors such as age (*p* = 0.002), gender (*p* < 0.001), educational level (*p* = 0.008) and worker category (*p* < 0.001) were associated with increased work-related musculoskeletal disorders, while work experience (*p* = 0.002) was associated with a decreased prevalence of back pain and discomfort. Females (AOR = 55.90; 95% CI [3.84, 814.54]), security personnel (AOR = 39.53, 95% CI [1.57, 996.00]), catering staff (AOR = 91.3295% CI [2.24, 3724.78]) and those who attained bachelor’s degrees (AOR = 73.25, 95% CI [1.46, 3682.39]), higher national diplomas (AOR = 93.49, 95% CI [1.28, 6848.04]) and national diplomas (AOR = 52.22; 95% CI [1.09, 2510.73]) had higher odds of experiencing WMSDs. Increased working experience was protective against experiencing lower back pain (AOR = 0.84; 95% CI [0.74, 0.95]).

**Discussion and conclusion:**

The prevalence of work-related musculoskeletal disorders was high among the participants. This was influenced by the nature of work as well as cultural factors.

**Recommendations:**

Occupational therapists and physiotherapists need to design focused ameliorative and health promotive interventions targeting at-risk populations in universities. Employers should consider developing wellness programs for workers and promoting healthy working environments.

**Supplementary Information:**

The online version contains supplementary material available at 10.1186/s12891-023-06900-1.

## Background

Musculoskeletal conditions are among the leading causes of disability worldwide, and chronic musculoskeletal conditions affect nearly the same percentage of the general population as chronic circulatory and respiratory conditions combined [1, 2]. Musculoskeletal disorders (MSDs) are the fourth highest contributor to years lived with disability [3] and among these work-related health problems are most common [4]. In Africa, the prevalence of WMSD is estimated to range from 15%- 93.5% [5].

Work-related musculoskeletal disorders (WMSDs) account for a large proportion of morbidity in the work force [6]. WMSDs are characterised as a group of painful disorders of muscles, tendons, joints and nerves, commonly affecting the neck, upper limbs and back, that result from engagement in work tasks or interaction with the work environment [7]. WMSDs are a result of a mismatch between the worker, the work task and the work environment. Several risk factors for the development of WMSDs have been identified, including repetitive work, awkward postures during work performance and discrepancies between the physical capacity of the human body and the physical requirements of the work task [8]. The incidence of specific WMSDs and reported discomfort in particular body regions is highly associated with specific work types [9–16]. This is mainly because workers in specific job types are engaged in work tasks that put specific body parts at higher risks of developing musculoskeletal disorders. For example, landscapers have a high prevalence of shoulder pain and discomfort related to their posturing during work performance [16], while nurses report a higher prevalence of lower back pain [9, 17]. However, it should be acknowledged that apart from these work performance related factors, multiple other psychological and ergonomic factors contribute to the increased incidence and high prevalence of WMSDs [17–21].

WMSDs are a major public health concern and frequently lead to temporary or permanent work incapacity. Musculoskeletal pain symptoms are the main contributor to absenteeism among workers [14, 22, 23]. Workers may frequently absent themselves from work or take sick leave, leading to shortages in the available labour [24, 25]. This has been linked to significant health costs and decrease in productivity leading to losses for companies [26, 27]. In more chronic cases, WMSDs may result in permanent disability and loss of employment and lead to considerable costs for the public health system [28–30].

The prevalence of WMSDs has been studied among various university populations in countries such as Malaysia, Ethiopia and Nigeria [16, 31, 32]. However, there is a paucity of evidence on WMSDs among university workers in Zimbabwe. This study stems from anecdotal reports from clinicians at the University of Zimbabwe Faculty of Medicine and Health Sciences (UZFMHS) Rehabilitation Clinic, who estimate that up to 60% of their clientele, mostly non-academic workers at the university, complain of musculoskeletal pain. This study therefore sought to determine the prevalence of WMSDs and associated factors among UZFMHS non-academic workers. Non-academic workers offer support services within the university, that ensure that the business of teaching and learning is conducted smoothly. These include administrative tasks, library services, technical support, food services, janitorial services and security services. This paper is the first of two and focuses mainly on the prevalence of WMSDs in the targeted population as well as its associated sociodemographic factors. In the second paper we focus on the associated ergonomic risk factors.

## Methods

### Study design

The study used a cross-sectional analytical design to determine the prevalence of work-related musculoskeletal disorders and associated factors among non-academic workers at the UZFMHS.

### Sampling and sample size

Participants were selected using proportional stratified sampling. Participants were stratified according to their job categories. These categories included administrative staff, security personnel, library staff, catering staff, drivers, cleaning staff and technicians. Calculations for the ratio of participants for each category were performed. Although, originally, the plan was to randomly select the participants in each category, the research team was unable to access a full register of all non-academic staff. Therefore, convenience sampling was used to select participants across all categories, until the sample size was reached.

All non-academic workers with at least 9 months of employment at UZFHMS were eligible for the study. However, all pregnant females or those who were 3-months postnatally, or workers who had an operation or had suffered an injury in the past year, which was not work-related, were excluded from the study.

The hypergeometric approximation calculation was used to determine the minimum sample size [33, 34].

$$n=\frac{{\mathrm{Z}}^{2}\mathrm{Np}(1-\mathrm{p})}{{\mathrm{\alpha }}^{2}\left(\mathrm{N}-1\right)+{\mathrm{Z}}^{2}\mathrm{p}(1-\mathrm{p})}$$

*where;* $$Z=1.96$$$$N=140$$ (total number of non-academic workers at UZFHMS) [35]

$$\alpha=0.05$$

$$p=0.7$$

$$=\frac{{(1.96)}^{2}(141)(0.7)(0.3)}{{(0.005)}^{2}(140)+{(1.96)}^{2}(0.7)(0.3)}$$

$$=\frac{\mathrm{113,749776}}{1.156736}$$

$$\mathrm{n}=98.336$$

$$\therefore \mathrm{n}=98$$

The minimum sample size required was 98. The authors added 10% of the minimum sample size to account for subject attrition, incomplete forms and non-response. A total of 108 participants were targeted for selection.

### Data collection and analysis

Data collection was done in January 2021. As the study was conducted during the 2019 Coronavirus (COVID-19) pandemic, the researchers adhered to the World Health Organisation and Ministry of Health and Child Care protocols during the data collection process. Both researchers and participants masked up, sanitised their hands and practiced social distancing.

The participants were requested to respond to a self-administered survey to determine the presence of WMSDs among the UZFMHS non-academic workers and the possible risk factors for WMSDs. The participants received a printed version of the questionnaire and were requested to complete the form at their own time. The researchers followed up and collected the completed questionnaires after five working days. Before responding to the study questionnaire, participants were asked to sign consent forms, in which their rights were articulated. The study questionnaire was adopted from the Nordic Musculoskeletal Questionnaire (NMQ) [36] by expanding the sociodemographic section and adding a section on ergonomic factors. The NMQ has been reported as a valid and reliable screening tool for musculoskeletal disorders [37]. The study questionnaire had three sections: section A had 5 items, and collected socio-demographic details including age, work experience, type of work et cetera; section B had 18 items and collected information on the body regions that experienced pain 12-months and 3-months prior; and section C had 15 items on the ergonomic factors that participants engage in at work. The final questionnaire was checked for content validity by a panel of occupational therapists with expertise in work rehabilitation, ergonomics and musculoskeletal disorders.

The Statistical Package for Social Sciences (SSPS) version 24 was used to analyse the data. Descriptive statistics, including frequencies and percentages, were calculated for socio-demographics including age, gender, level of education, years of work experience and work designation. The prevalence of WMSDs was calculated from the frequency of responses disaggregated by body region. Tests of association, such as Chi-squared and Mann–Whitney U tests, were computed to determine the relationship of various factors with the prevalence of WMSDs. Furthermore, we used multivariate logistic regression to determine which sociodemographic factors influenced the odds of developing WMSDs.

## Results

One hundred and eight questionnaires were distributed to the participants. Out of 108 questionnaires, only 100 completed questionnaires were returned, yielding a response rate of 92.6%. The mean age of the participants was 45.87 years (SD = 6.678), with participants ranging from 30 to 60 years of age. The mode age was 55 years (*n* = 8, 8%), with a median age of 46 years. Among the respondents were 54 (54%) were males and 46 (46%) were females. The sample consisted of administrative staff (*n* = 25, 25%), security staff (*n* = 22, 22%), catering staff (*n* = 18, 18%), technicians (*n* = 18, 18%), cleaning staff (*n* = 8, 8%), drivers (*n* = 6, 6%) and librarians (*n* = 5, 5%). The majority of participants (*n* = 29, 29%) had General Certificate of Education Ordinary Level (GCE O`Level) as their highest qualification, while 20 (20%) held a Higher National Diploma (HND), 18(18%) held a National Diploma (ND), 18 (18%) held a Bachelor’s Degree and 10 (10%) held a Master’s Degree. A small fraction had attained General Certificate of Education Advanced level (GCE A`Level) (*n* = 2, 2%) and National Certificates (NC) (*n* = 3, 3%). Table 1 summarises the participants` socio-demographic characteristics.

**Table 1:** Demographic characteristics of participants

VARIABLE	
**AGE**	**Years**
Mean (SD)^a^	45.87 (SD = 6.678)
Median	46
Mode	55
Minimum	30
Maximum	60
**FREQUENCY n (%)**	
**GENDER**	
Female	46 (46)
Male	54(54)
**WORKER CATEGORY**	
Security staff	22(22)
Catering staff	16(16)
Library staff	5(5)
Cleaning staff	8(8)
Technicians	18(18)
Drivers	6(6)
Clerical staff	25(25)
**HIGHEST QUALIFICATION**	
Master’s Degree	10(10)
Bachelor’s Degree	18(18)
Higher National Diploma	20(20)
National Diploma	18(18)
National Certificate	3(3)
Advanced Level	2(2)
Ordinary Level	29(29)

^a^*SD means* Standard deviation

### Prevalence of WMSDs among UZFMHS non-academic workers

The participants reported having experienced pain or discomfort in one or more body parts within both the past 12 months and 3 months. The majority of participants reported having experienced pain or discomfort in the lower back (*n* = 75, 75%) within the past 12 months. Other regions with a reported 12-month prevalence of pain or discomfort were the wrists/hands (*n* = 69, 69%), upper back (*n* = 48, 48%), ankles (*n* = 46, 46%), knees (*n* = 40, 40%), shoulders (*n* = 38, 38%), thighs/hips (*n* = 37, 37%), neck (*n* = 36, 36%) and elbows (*n* = 19, 19%). The participants also reported having experienced pain and discomfort in the lower back (*n* = 72, 72%), wrists/hands (*n* = 60, 60%), upper back (*n* = 46. 46%), ankles (*n* = 46, 46%), knees (*n* = 40, 40%), shoulders (*n* = 38, 38%), neck (*n* = 33, 33%), thighs/hips (*n* = 34, 34%) and elbows (*n* = 18, 18%) in the past 3-months (Table 2).

**Table 2:** Twelve-month and 3-month prevalence of WMSDs among workers at the UZFMHS

Experience of pain in		Neck	Shoulders	Elbows	Wrists/Hands	Upper back	Lower back	Thighs\Hips	Knees	Ankles
**Frequency, n (%) in the past 12 months**	YES	36 (36.0)	38 (38.0)	19 (19.0)	69 (69.0)	48 (48.0)	75 (75.0)	37 (37.0)	40 (40.0)	46 (46.0)
	NO	64 (64.0)	62 (62.0)	81 (81.0)	31 (31.0)	52 (52.0)	25 (25.0)	63 (63.0)	60 (60.0)	54 (54.0)
**Frequency, n (%) in the past 3 months**	YES	33 (33.0)	38 (38.0)	18 (18.0)	60 (60.0)	46 (46.0)	72 (72.0)	34 (34.0)	40 (40.0)	46 (46.0)
	NO	67 (67.0)	62 (62.0)	82 (82.0)	40 (40.0)	54 (54.0)	28 (28.0)	66 (66.0)	60 (60.0)	54 (54.0)

### Prevalence of WMSD and associated sociodemographic factors

#### Age

Tables 3 and 4 show how age was associated with the 12-month and 3-month prevalence of WMSDs respectively. The majority (*n* = 17, 85%) of the participants aged 30–39 years reported having experienced pain or discomfort in the wrists in the past 12 months. Those in the > 50 years age group mainly complained of the lower back (*n* = 26, 81.25%) in the same time period (Table 3). Within the past 3 months, the highest prevalence of pain or discomfort was in the wrist or hand (*n* = 16, 80%) among participants in the 30–39-year age group, while the 40–49 years and  > 50 years age groups mainly reported experiencing pain or discomfort in the lower back [(*n* = 34, 70.83%) and (*n* = 25, 78.13%) respectively] (Table 4). Age was associated with experiencing pain and discomfort in the wrist/hand (*p* = 0.008) and the ankles (*p* = 0.047) in the past 12 months. Age was also associated with experiencing pain and discomfort in the wrist/hands (*p* = 0.002), while an increase in age was associated with pain in the lower back (*p* < 0.05) within the last 3 months.

**Table 3:** Association of 12-month prevalence of WMSD among non-academic workers and age

Age (Years)	Response	Neck	Shoulders	Elbows	Wrists/Hands	Upper back	Lower back	Thighs/Hips	Knees	Ankles
**30–39**	Yes	3(15.00)	11(55.00)	5(25.00)	17(85.00)	8(40.00)	14(70.00)	7(35.00)	9(45.00)	11(55.00)
	No	17(85.00)	9(45.00)	15(75.00)	3(15.00)	12(60.00)	6(30.00)	13(65.00)	11(55.00)	9(45.00)
**40–49**	Yes	19(39.58)	13(27.08)	6(12.50)	28(58.33)	26(54.17)	36(75.00)	23(47.92)	20(41.67)	25(52.08)
	No	29(60.42)	35(72.92)	42(87.50)	20(41.67)	22(45.84)	12(25.00)	25(52.08)	28(58.33)	23(47.92)
** > 50**	Yes	14(43.75)	14(43.75)	8(25.00)	17(53.13)	14(43.75)	26(81.25)	12(37.50)	11(34.38)	10(31.25)
	No	18(56.25)	18(56.25)	24(75.00)	15(46.88)	18(56.25)	6(18.75)	20(62.50)	21(65.63)	22(68.75)
**Mann–Whitney test** ***P* value**		0.263	0.795	0.801	0.008	0.456	0.814	0.421	0.234	0.047

**Table 4:** Association of 3-month prevalence of WMSD among non- academic workers at UZFHMS and age

Age (Years)	Response	Neck	Shoulders	Elbows	Wrists/Hands	Upper Back	Lower Back	Thighs/Hips	Knees	Ankles
**30–39**	Yes	3(15.00)	11(55.00)	5(25.00)	16(80.00)	8(40.00)	14(70.00)	7(35.00)	9(45.00)	11(55.00)
	No	17(85.00)	9(45.00)	15(75.00)	4(20.00)	12(60.00)	6(30.00)	13(65.00)	11(55.00)	9(45.00)
**40–49**	Yes	18(37.50)	13(27.08)	6(12.50)	27(56.25)	24(50.00)	34(70.83)	19(39.58)	20(41.67)	25(52.08)
	No	30(62.50)	35(72.92)	42(87.50)	21(43.75)	24(50.00)	14(29.17)	29(60.42)	28(58.33)	23(47.92)
** > 50**	Yes	12(37.50)	14(43.75)	7(21.86)	16(50.00)	14(43.75)	25(78.13)	12(37.50)	11(34.38)	10(31.25)
	No	20(62.50)	18(56.25)	24(78.13)	16(50.00)	18(56.25)	7(21.88)	20(62.50)	21(65.63)	22(68.75)
**Mann–Whitney test** ***P* value**		0.143	0.765	0.570	0.002	0.463	< 0.001	0.446	0.296	0.061

#### Gender

The association of gender and the prevalence of WMSDs is presented in Table 5. The highest prevalence of reported pain or discomfort in the past 12 months was in the lower back, which was reported by almost all females (*n* = 43, 93.5%) and more than half of males (*n* = 32, 59.3%). The lower back was again reported to have the highest 3-month prevalence for both males (*n* = 31, 54.4%) and females (*n* = 41, 89.1%). Gender was associated with pain and discomfort in the lower back at both 12 months (*p* < 0.05) and 3 months (*p* = 0.001), being more prevalent in females. On the other hand, male gender was associated with shoulder pain or discomfort (*p* = 0.038) at 3 months.

**Table 5:** Twelve-month and 3-month prevalence of WMSD among non-academic workers at UZFHMS and associated gender

Body region	12-month prevalence against GENDER					3-month prevalence against GENDER			
		Male, n(%)	Female, n(%)	Fischer exact			Male, n(%)	Female, n(%)	Fischer exact
**Neck**	Yes	16(29.6)	17(37.0)	0.524	Yes		16(29.6)	20(43.5)	0.210
	No	38(70.4)	29(63.0)		No		38(70.4)	26(56.5)	
**Shoulders**	Yes	25(46.3)	13(28.3)	0.098	Yes		26(48.1)	12(26.1)	0.038
	No	29(53.7)	33(71.7)		No		28(51.9)	34(73.9)	
**Elbows**	Yes	11(20.4)	7(15.2)	0.605	Yes		11(20.4)	8(17.4)	0.801
	No	43(79.6)	39(84.8)		No		43(79.6)	38(82.6)	
**Wrists/Hands**	Yes	31(57.4)	30(65.2)	0.538	Yes		30(55.6)	30(65.2)	0.413
	No	23(42.6)	16(34.8)		No		24(44.4)	16(34.8)	
**Upper** **Back**	Yes	23(42.6)	25(54.3)	0.316	Yes		21(38.9)	25(54.3)	0.159
	No	31(54.4)	21(45.7)		No		33(61.1)	21(45.7)	
**Lower** **Back**	Yes	32(59.3)	43(93.5)	< 0.001	Yes		31(54.4)	41(89.1)	0.001
	No	22(40.7)	3(6.50)		No		23(45.6)	5(10.9)	
**Thighs/Hips**	Yes	16(29.6)	21(45.7)	0.145	Yes		15(27.8)	19(41.3)	0.601
	No	38(70.4)	25(54.3)		No		39(72.2)	27(58.7)	
**Knees**	Yes	20(37.0)	20(43.5)	0.544	Yes		20(37.0)	20(43.5)	0.544
	No	34(63.0)	26(56.5)		No		34(63.0)	26(56.5)	
**Ankles**	Yes	25(46.3)	21(45.7)	1.000	Yes		25(46.3)	21(45.7)	1.000
	No	29(53.7)	25(54.3)		No		29(53.7)	25(54.3)	

#### Years of working experience

Increasing years of working experience was associated with a significant decrease in experiencing pain or discomfort in the upper back (*p* = 0.002) and lower back (*p* = 0.002) within the last 12 months. A decreased prevalence of pain or discomfort within the last 3 months in the wrist/hands (*p* = 0.033), upper back (*p* = 0.003), lower back (*p* = 0.011) and thigh/hips (*p* < 0.001) was also associated with increasing years of working experience. The highest 12-month prevalence of pain or discomfort was reported in the wrists by all the participants with > 31 years of working experience (*n* = 4, 100%), while 86.67% (*n* = 26) of those with 2–11 years of working experience reported experiencing pain or discomfort in the lower back in the same time period (Table 6). Participants with 12–21 years of working experience mostly reported experiencing lower back pain or discomfort (*n* = 42, 79.25%) in the past 12 months. The highest 3-month prevalence of pain and discomfort was reported in the lower back (*n* = 41, 77.36%) among those with 12–21 years of working experience, while those in the 2–11 and > 31 years of working experience ranges mainly reported experiencing pain or discomfort in the wrist/hands (*n* = 25, 85%) and lower back (*n* = 24, 80%); and neck, shoulder and wrists (*n* = 3, 75%) (Table 7).

**Table 6:** Twelve-month prevalence of WMSDs among non-academic workers at UZFHMS and associated years of working experience

**Years**		**Neck** **n(%)**	**Shoulders** **n(%)**	**Elbows** **n(%)**	**Wrists/ Hands** **n(%)**	**Upper back n(%)**	**Lower back** **n(%)**	**Thighs/ Hips** **n(%)**	**Knees** **n(%)**	**Ankles** **n(%)**
**2–11**	Yes	10(33.33)	12(40.00)	7(23.33)	24(80.00)	19(63.33)	26(86.67)	13(43.33)	15(50.00)	17(56.67)
	No	20(66.67)	18(60.00)	23(76.67)	6(20.00)	11(36.67)	4(13.33)	17(56.67)	15(50.00)	13(43.33)
**12–21**	Yes	14(26.42)	18(33.96)	9(16.98)	29(54.72)	26(49.06)	42(79.25)	20(37.74)	20(37.74)	24(45.28)
	No	39 (73.58)	35(66.04)	44(83.02)	24(45.28)	27(50.94)	11(20.75)	33(62.26)	33(62.26)	29(54.72)
**22–31**	Yes	6(46.15)	5(38.46)	1(7.69)	4(30.77)	2(15.38)	6(46.15)	4(30.77)	5(38.46)	4(30.77)
	No	7(53.85)	8(61.54)	12(92.31)	9(69.23)	11(85.52)	7(53.85)	9(69.23)	8(61.54)	9(69.23)
** > 31**	Yes	3(75.00)	3(75.00)	1(25.00)	4(100.00)	1(25.00)	1(25.00)	0(0.00)	0(0.00)	1(25.00)
	No	1(25.00)	1(25.00)	3(75.00)	0(0.00)	3(75.00)	3(75.00)	4(100.00)	4(100.00)	3(75.00)
**Mann- Whitney test *P* value**		0.129	0.795	0.410	0.134	0.002	0.002	0.233	0.604	0.338

**Table 7:** 3-month prevalence of WMSDs among non-academic workers at UZFHMS and associated years of working experience

**Years**		**Neck** **n(%)**	**Shoulders** **n(%)**	**Elbows** **n(%)**	**Wrists/ Hands** **n(%)**	**Upper back n(%)**	**Lower back** **n(%)**	**Thighs/ Hips** **n(%)**	**Knees** **n(%)**	**Ankles** **n(%)**
**2–11**	Yes	10(33.33)	12(40.00)	7(23.33)	25(85.00)	18(60.00)	24(80.00)	11(36.67)	13(43.33)	17(56.67)
	No	20(66.67)	18(60.00)	23(76.67)	5(15.00)	12(40.00)	6(20.00)	19(63.33)	17(56.67)	13(43.33)
**12–21**	Yes	17(32.08)	18(33.96)	10(18.87)	28(52.83	25(47.17)	41(77.36)	19(35.85)	22(41.51)	24(45.28)
	No	36(67.92)	35(66.04)	43(81.13)	25(47.17)	28(52.83)	13(22.64)	34(64.15)	31(58.49)	29(54.72)
**22–31**	Yes	6 (41.15)	5 (38.46)	1(7.69)	4(30.77)	2(15.38)	6(46.15)	4(30.77)	5(38.46)	4(30.77)
	No	7(53.85)	8(61.54)	12(92.31)	9(69.23)	11(84.62)	7(53.85)	9(69.23)	8(61.54)	9(69.23)
** > 31**	Yes	3(75.00)	3(75.00)	1(25.00)	3(75.00)	1(25.00)	1(25.00)	0(0.00)	0(0.00)	1(25.00)
	No	1(25.00)	1(25.00)	3(75.00)	1(25.00)	3(75.00)	3(75.00)	4(100.00)	4(100.00)	3(75.00)
**Mann- Whitney test *P* value**		0.080	0.659	0.523	0.033	0.003	0.011	0.813	0.913	0.212

#### Highest qualification

Table 8 shows that wrist/hand pain had the highest 12-month prevalence of 90% (*n* = 18) among those with HNDs and 88.89% (*n* = 16) among those with NDs. The Pearson Chi-square test showed a significant association between the 12-month prevalence of WMSD and the highest qualification in the neck, wrists and ankles. (Table 8). The highest qualification was also associated with pain or discomfort in the neck (*p* = 0.01) and wrist/hand (*p* = 0.008) within the last 3 months (Table 9).

**Table 8:** Twelve-month prevalence of WMSD among non-academic workers at UZFHMS and associated highest qualification

**REGION**		**Highest Qualification n(%)**							**Chi-square test, χ** **(*p* value)**
		Master’s	Bachelor’s	HND^a^	ND^a^	NC^a^	GCE A level^a^	GCE O level^a^	
**Neck**	Yes	6 (60.00)	6 (33.33)	8 (40.00)	8 (44.44)	2 (66.67)	1 (50.00)	2 (6.90)	15.544 (0.016)
	No	4 (40.00)	12 (66.67)	12 (60.00)	10 (55.56)	1 (33.33)	1 (50.00)	27 (93.3)	
**Shoulders**	Yes	4 (40.00)	9 (50.00)	10 (50.00)	4 (22.22)	1 (33.33)	1 (50.00)	9 (31.03)	4.989 (0.545)
	No	6 (60.00)	9 (50.00)	10 (50.00)	14 (77.78)	2 (66.67)	1 (50.00)	20 (68.97)	
**Elbows**	Yes	2 (20.00)	5 (27.78)	6 (30.00)	3 (20.00)	0 (0.00)	1 (50.00)	1 (3.45)	9.372 (0.154)
	No	8 (80.00)	13 (72.22)	14 (70.00)	15 (80.00)	3 (100.00)	1 (50.00)	28 (96.55)	
**Wrists/Hands**	Yes	3 (30.00)	12 (66.67)	18 (90.00)	16 (88.89)	2 (66.67)	1 (50.00)	10 (34.48)	23.840 (0.001)
	No	7 (70.00)	6 (33.33)	2 (20.00)	2 (11.11)	1 (33.33)	1 (50.00)	19 (65.52)	
**Upper** **Back**	Yes	2 (20.00)	9 (50.00)	9 (45.00)	10 (55.56)	2 (66.67)	0 (0.00)	16 (55.17)	6.516 (0.368)
	No	8 (80.00)	9 (50.00)	11 (55.00)	8 (44.44)	1 (33.33)	2 (100.00)	13 (44.83)	
**Lower** **Back**	Yes	5 (50.00)	14 (77.78)	15 (75.00)	15 (80.00)	2 (66.67)	1 (50.00)	23 (79.31)	5.139 (0.526)
	No	5 (50.00)	4 (22.22)	5 (25.00)	3 (20.00)	1 (33.33)	1 (50.00)	6 (20.69)	
**Thighs/Hips**	Yes	4 (40.00)	4 (22.22)	7 (35.00)	8 (44.44)	2 (66.67)	0 (0.00)	12 (48.38)	4.733 (0.578)
	No	6 (60.00)	14 (77.78)	13 (65.00)	10 (55.56)	1 (33.33)	2 (100.00)	17 (58.62)	
**Knees**	Yes	4 (40.00)	4 (22.22)	7 (35.00)	10 (55.56)	2 (66.67)	0 (0.00)	13 (44.83)	6.987 (0.330)
	No	6 (60.00)	14 (77.78)	13 (65.00)	8 (44.44)	1 (33.33)	2 (100.00)	16 (55.17)	
**Ankles**	Yes	2 (20.00)	6 (33.33)	6 (30.00)	14 (77.78)	2 (66.67)	0 (0.00)	16 (55.17)	16.465 (0.011)
	No	8 (80.00)	12 (66.67)	14 (70.00)	4 (22.22)	1 (33.33)	2 (100.00)	13 (44.83)	

^a^*HND* Higher national diploma, *ND* National diploma, *NC* National certificate, *GCE A level* General certificate of education advanced level, *GCE O level* General certificate of education ordinary level

**Table 9:** 3-month prevalence of WMSD among non-academic workers at UZFHMS and associated highest qualification

**Body region**		**Highest qualification n(%)**							**Chi-square test, χ** **(*p* value)**
		Master’s	Bachelor’s	HND^a^	ND^a^	NC^a^	GCE A level^a^	GCE O level^a^	
**Neck**	Yes	6 (60.00)	7 (38.89)	9 (45.00)	9 (50.00)	2 (66.67)	1 (50.00)	2 (6.90)	16.855 (0.010)
	No	4 (40.00)	11 (61.11)	11 (55.00)	9 (50.00)	1 (33.33)	1 (50.00)	27 (93.10)	
**Shoulder**	Yes	4 (40.00)	9 (50.00)	11 (55.00)	4 (22.22)	1 (33.33)	1 (50.00)	8 (27.59)	6.957 (0.325)
	No	6 (60.00)	9 (50.00)	9 (45.00)	14 (77.78)	2 (66.67)	1 (50.00)	21 (72.41)	
**Elbows**	Yes	2 (20.00)	5 (27.28)	7 (35.00)	3 (20.00)	0 (0.00)	1 (50.00)	1 (3.45)	10.808 (0.094)
	No	8 (80.00)	13 (72.22)	13 (65.00)	15 (80.00)	3 (100.00)	1 (50.00)	28 (96.55)	
**Wrist/Hands**	Yes	4 (40.00)	12 (66.67)	16 (80.00)	15 (80.00)	2 (66.67)	1 (50.00)	10 (34.48)	17.423 (0.008)
	No	6 (60.00)	6 (33.33)	4 (20.00)	3 (20.00)	1 (33.33)	1 (50.00)	19 (65.52)	
**Upper** **Back**	Yes	2 (20.00)	5 (27.28)	6 (30.00)	3 (20.00)	0 (0.00)	1 (50.00)	1 (3.45)	6.065 (0.416)
	No	8 (80.00)	13 (72.22)	14 (70.00)	15 (80.00)	3 (100.00)	1 (50.00)	28 (96.55)	
**Lower** **Back**	Yes	4 (40.00)	13 (72.22)	15 (75.00)	15 (80.00)	2 (66.67)	1 (50.00)	22 (75.86)	7.053 (0.316)
	No	6 (60.00)	5 (27.28)	5 (25.00)	3 (20.00)	1 (33.33)	1 (50.00)	7 (24.14)	
**Thighs/Hips**	Yes	2 (20.00)	8 (44.44)	9 (45.00)	9 (50.00)	2 (66.67)	0 (0.00)	16 (55.17)	13.567 (0.329)
	No	8 (80.00)	10 (55.56)	11 (55.00)	9 (50.00)	1 (33.33)	2 (100.00)	13 (44.83)	
**Knees**	Yes	3 (30.00)	4 (22.22)	7 (35.00)	8 (44.44)	2 (66.67)	0 (0.00)	10 (34.48)	5.389 (0.495)
	No	7 (70.00)	14(77.78)	13 (65.00)	10 (55.56)	1 (33.33)	2 (100.00)	19 (65.52)	
**Ankles**	Yes	4 (40.00)	5 (27.28)	7 (35.00)	10 (55.56)	2 (66.67)	0 (0.00)	12 (41.38)	10.953 (0.090)
	No	6 (60.00)	13 (72.22)	13 (65.00)	8 (44.44)	1 (33.33)	2 (100.00)	17 (58.62)	

^a^*HND* Higher national diploma, *ND* National diploma, *NC* National certificate, *GCE A level* General certificate of education advanced level, *GCE O level* General certificate of education ordinary level

#### Worker category

All worker categories reported high 12-month and 3-month prevalence in two or more body regions. Table 10 shows the 12-month prevalence of WMSD according to worker category for the various body regions. Librarians reported the highest 12-month prevalence of WMSD, with all (*n* = 5, 100%) reporting having experienced lower back pain. A high 12-month prevalence of lower back pain was also reported among catering staff (*n* = 15, 93.80%), clerical staff (*n* = 22, 88.00%) and security personnel (*n* = 18, 81.80%). The same worker categories also reported a 3-month prevalence of lower back pain above 80% (Table 11).

**Table 10:** Twelve-month prevalence of WMSD among UZFMHS non-academic workers and associated worker category (designation)

Pain experience on specific body region	Response	WORKERCATEGORY							Chi-square test, χ (p value)
		Security n(%)	Catering n(%)	Library n(%)	Cleaning staff n(%)	Technicians n(%)	Drivers n(%)	Clerical staff n(%)	
**Neck**	Yes	3 (13.60)	0 (0.00)	4 (80.00)	0 (0.00)	10 (55.60)	4 (66.70)	12 (48.00)	30.490 (0.000)
	No	19 (86.4)	16(100.00)	1(20.00)	8(100.00)	8 (44.40)	2(33.30)	13(52.00)	
**Shoulder**	Yes	7 (31.80)	8 (50.00)	1 (20.00)	4 (50.00)	12 (66.70)	1 (16.70)	5 (20.00)	13.387 (0.063)
	No	15 (68.20)	8 (50.00)	4 (80.00)	4 (50.00)	6 (33.30)	5 (83.30)	20 (80.00)	
**Elbow**	Yes	1 (4.50)	3 (18.80)	1 (20.00)	0 (0.00)	6 (33.30)	1 (16.70)	6 (24.00)	9.042 (0.250)
	No	21 (95.50)	13 (81.20)	4 (80.00)	8(100.00)	12 (66.70)	5 (83.30)	19 (76.00)	
**Wrist/Hand**	Yes	6 (27.30)	12 (75.00)	3 (60.00)	5 (40.00)	14 (77.80)	4 (66.70)	17 (68.00)	14.741 (0.039)
	No	16 (72.70)	4 (25.00)	2 (40.00)	3 (60.00)	4 (22.20)	2 (33.30)	8 (32.00)	
**Upper back**	Yes	14 (63.60)	7 (43.70)	1 (20.00)	3 (37.50)	4 (22.20)	2 (33.30)	17 (68.00)	13.670 (0.057)
	No	8 (36.40)	9 (56.30)	4 (80.00)	5 (62.50)	14 (77.80)	4 (66.70)	8 (32.00)	
**Lower back**	Yes	18 (81.80)	15 (93.80)	5 (100.00)	5 (62.50)	6 (22.20)	4 (66.70)	22 (88.00)	25.501 (0.001)
	No	4 (18.20)	1 (6.20)	0 (0.00)	3 (37.50)	12 (77.80)	2 (33.30)	3 (12.00)	
**Thighs/Hips**	Yes	9 (40.90)	11 (68.80)	2 (40.00)	4 (50.00)	0 (0.00)	0 (0.00)	11 (44.00)	22.327 (0.002)
	No	13 (69.10)	5 (31.20)	3 (60.00)	4 (50.00)	18 (100.00)	6(100.00)	14 (66.00)	
**Knees**	Yes	10(45.50)	14 (87.50)	2 (40.00)	2 (25.00)	1 (5.60)	0 (0.00)	11 (44.00)	29.769 (0.000)
	No	12 (54.50)	2 (12.50)	3 (60.00)	6 (75.00)	17(94.40)	6 (100.00)	14 (66.00)	
**Ankles**	Yes	14 (63.60)	13 (81.20)	2 (40.00)	3 (37.50)	1 (5.60)	3 (50.00)	10 (40.00)	24.324 (0.001)
	No	8 (36.40)	3 (18.80)	3 (60.00)	5 (62.50)	17(94.40)	3 (50.00)	15 (60.00)

**Table 11:** 3-month prevalence of WMSD among UZFMHS non-academic workers and associated worker category (designation)

**Pain experience on specific body region**	**Response**	**WORKERCATEGORY**							**Chi-square test, χ** (*p* value)
		Security n(%)	Catering n(%)	Library n(%)	Cleaning staff n(%)	Technicians n(%)	Drivers n(%)	Clerical staff n(%)	
**Neck**	Yes	3 (13.60)	2 (12.50)	4(80.00)	0 (0.00)	10 (55.60)	4 (66.70)	12 (48.00)	26.91 (0.001)
	No	19 (86.40)	14 (87.50)	1(20.00)	8 (100.00)	8 (44.40)	2 (33.30)	13 (52.00)	
**Shoulder**	Yes	6 (27.30)	9 (56.25)	1(20.00)	4 (50.00)	12 (66.70)	1 (16.70)	5 (20.00)	15.388 (0.031)
	No	16 (73.70)	7 (43.75)	4(80.00)	4 (50.00)	6 (33.30)	5 (83.30)	20 (80.00)	
**Elbow**	Yes	1 (4.50)	4 (25.00)	1(20.00)	0 (0.00)	6 (33.30)	1 (16.70)	6 (24.00)	9.111 (0.245)
	No	21 (95.50)	12 (75.00)	4(80.00)	8 (100.00)	12 (66.70)	5 (83.30)	19 (76.00)	
**Wrist/Hand**	Yes	6 (27.30)	11 (68.75)	3(60.00)	5 (62.50)	14 (77.80)	4 (66.70)	17 (68.00)	3.664 (0.057)
	No	16 (72.7)	5 (21.25)	2(40.00)	3 (33.30)	4 (22.20)	2 (33.30)	8 (32.00)	
**Upper back**	Yes	14 (63.60)	6 (37.50)	1(20.00)	3 (37.50)	4 (22.20)	1 (16.70)	17 (68.00)	16.021 (0.025)
	No	8 (36.4)	10 (62.50)	4(80.00)	5 (62.50)	14 (77.80)	5 (83.30)	8 (32.00)	
**Lower back**	Yes	18 (81.80)	15 (93.80)	5(100.00)	5 (62.50)	5 (27.28)	3 (50.00)	21 (84.00)	27.846 (0.000)
	No	4 (18.20)	1 (6.20)	0 (0.00)	3(33.30)	13 (72.22)	3 (50.00)	4 (16.00)	
**Thighs/Hips**	Yes	8 (36.40)	11 (68.80)	2(40.00)	3 (33.30)	0 (0.00)	0 (0.00)	10 (40.00)	21.553 (0.003)
	No	14 (63.60)	5 (31.20)	3(60.00)	5 (62.50)	18(100.00)	6 (100.00)	15 (50.00)	
**Knees**	Yes	10(45.50)	14 (87.50)	3(60.00)	1 (14.29)	2 (5.56)	0 (0.00)	10 (40.00)	29.969 (0.000)
	No	12 (54.50)	2 (12.50)	2(40.00)	7 (85.71)	16 (94.44)	6 (100.00)	15 (60.00)	
**Ankles**	Yes	14 (63.60)	12 (75.00)	2(40.00)	2 (25.00)	2 (5.56)	3 (50.00)	11 (44.00)	19.208 (0.008)
	No	8 (36.40)	4 (25.00)	3(60.00)	6 (75.00)	16 (94.44)	3 (50.00)	14 (66.00)	

Worker category was associated with a 12-prevalence of WMSDs in the neck (*p* < 0.001), wrist (*p* = 0.039), lower back (*p* = 0.001), thighs (*p* = 0.002), knees (*p* < 0.001) and ankles (*p* = 0.001). The worker category was also associated with the 3-month prevalence of pain and discomfort in the neck (*p* = 0.001), shoulder(*p* = 0.031), upper back (0.025), lower back (*p* < 0.001), thighs (0.003), knees (*p* < 0.001), and ankles (0.008).

### Sociodemographic factors and odds of developing WMSDs

Multivariate logistic regression was used to analyse the relationship between sociodemographic factors and WMSDS in the various body regions. Although all sociodemographic factors had some effect on the odds of experiencing WMSDs, only gender, highest qualification, worker category and work experience had a significant influence on the prevalence of WMSDs. Holding all other sociodemographic factors constant, being female increased the odds of experiencing neck pain in the past 12 months (AOR = 8.05; 95% CI [1.10, 58.85]) and lower back pain at both 12 months (AOR = 55.90; 95% CI [3.84, 814.54]) and 3-months (AOR = 38.44; 95% CI [2.99, 494.40]). The highest qualification increased the odds of lower back pain at 3 months. Having attained a bachelor’s degree increased the odds of lower back pain (AOR = 73.25, 95% CI [1.46, 3682.39]), and similarly, having an HND increased the odds of lower back pain (AOR = 93.49, 95% CI [1.28, 6848.04]). Participants with NDs also had increased odds of experiencing lower back pain at 3 months (AOR = 52.22; 95% CI [1.09, 2510.73]). Worker categories also increased the odds of WMSDs, with being part of the security personnel increasing the odds of experiencing lower back pain at both 12 months (AOR = 39.53, 95% CI [1.57, 996.00]) and 3 months (AOR = 22.35, 95% CI [1.05, 476.20]), while the odds of knee pain at 12 months increased among catering staff (AOR = 91.3295% CI [2.24, 3724.78]). Work experience was protective against experiencing lower back pain, decreasing the odds at 3 months (AOR = 0.88, 95% CI [0.79, 0.98]) and at 12 months (AOR = 0.84; 95% CI [0.74, 0.95]).

## Discussion

In the current study, non-academic workers at the University of Zimbabwe Faculty of Medicine and Health Sciences reported experiencing pain and discomfort across all body regions in the past year and the preceding 3 months before the study. A high prevalence of WMSDs was reported in the lower back as well as the wrists or hands, with more than 60% of the sample reporting having experienced discomfort or pain. These results are consistent with those of studies conducted with non-academic workers at institutions of higher education in Ethiopia and Nigeria [31, 32]. In Ethiopia, the prevalence of WMSDs was reported to be 52.3% among university cleaners [31] while it was as high as 71.9% among office workers in Nigerian higher education institutions [32]. The prevalence of WMSDs was associated with various sociodemographic factors such as age, gender, work experience, highest qualification and worker categories. Younger age was associated with WMSD in the wrist and hands, while older age was associated with lower back pain. This is probably because younger workers tend to be assigned or take on tasks that expose them to overexertion and straining of musculoskeletal components [38]. On the other hand, older workers tend to experience more lower back pain due to natural vertebral disc degeneration with advancing age [39, 40]. Female gender was associated with experiencing lower back pain, while men experienced shoulder pain and discomfort. This might possibly be due to ergonomic factors as well as other cultural roles that require heavy lifting [32, 41]. Females’ higher likelihood of experiencing lower back pain may be the related to experiences of lower back pain resulting from pregnancy and in the post-partum period, which can be enduring [42, 43]. Increasing work experience was significantly associated with a decreased prevalence of WMSD in the past year. Years of work experience were protective, decreasing the odds of experiencing lower back pain by 12–16%. This is in contrast to studies at institutes of higher education in Nigeria and Saudi Arabia [32, 44], which reported an association of increasing WMSDs with increased work experience. This difference might be attributed to cultural nuances. In Saudi Arabia WMSDs increased with years of working experience because the workers also increased in age, developing associated degenerative processes, and accumulated stress to musculoskeletal structures as the years increased. In the Zimbabwean work context, older workers also experience age-related degenerative processes and, with increasing work experience, cumulative stress on musculoskeletal structures. However, tendencies are that older workers, with more work experience, do not engage in heavy work, leaving it for younger less experienced workers, as it is culturally expected that elders should not engage in heavy work when younger people are available. Furthermore, it could be that those with more working experience had mastered utilising ergonomic practices in executing their work tasks [45], although Okezue and colleagues [32] argue that increased work experience does not imply a more effective ergonomic response.

In this study, educational qualifications were associated with the prevalence of WMSD. This is due to the nature of jobs that require a certain level of education. As a result, there was also a similar association with worker categories. The findings are consistent with other studies conducted in Ethiopia, India, Italy, Malaysia, Nigeria and Pakistan [12, 13, 15, 31, 32, 46, 47] on various non-academic worker categories similar to those reported in the current study. Lower back pain was associated with work categories that spent large amounts of time seating, standing or engaged in heavy lifting such as security, library staff, catering staff and clerical staff. Security personnel had significantly higher odds of experiencing lower back pain while catering staff had higher odds of experiencing knee pain. Security duties placed personnel in situations that required them to stand or sit for extended periods of time. Saddique and colleagues [46] concur that security personnel experience a higher prevalence of lower back pain because they spend long hours standing or in static postures. Similarly, catering staff experienced pain and discomfort in the lower limb joints, and they spent most of their time standing. Additionally, catering staff also experienced pain and discomfort in the joints of the upper limbs, as they had to carry heavy ingredients and take out waste, as well as stir large pots during meal preparation. These findings are consistent with findings by Dempsey and Filiaggi [48] and Giorgianni and colleagues [12] in catering workers. The nature of the work done influences the strain and stress on musculoskeletal structures in specific body regions [15, 49]. For example, cleaners experience lower back pain and wrist pain because of the postures they assume during navigation of the cleaning environment and execution of the cleaning tasks [31].

### Strengths and limitations

Our present study sought to determine the association between sociodemographic factors and the prevalence of WMSD. Through multivariate logistic regression, the study further elucidated the factors that increased or decreased the odds of experiencing WMSDs. The study was conducted during the COVID-19 pandemic and authors were restricted in accessing measurement equipment such as scales and rules as well as in the nature of their interactions with participants. As a result, important factors like the body mass index (BMI) were not measured. The relationship between BMI and WMSDs is well researched and documented [50–54]. Furthermore, we could not randomly sample our participants because we were restricted access to worker registers by faculty administration. This lack of randomisation could have introduced some bias in our results. However, proportional stratification ensured a representative sample where all worker categories were accounted for to reflect the population under study. Although content validity of the final data collection instrument was checked by a panel of expert occupational therapists, the authors acknowledge that the psychometric properties of the Nordic Musculoskeletal Questionnaire in the setting and population. A pilot study was not conducted because of the small study population, limited available time for the study and COVID-19 restrictions. The study was conducted as part of an undergraduate research project at the height of the COVID-19 pandemic. However, to improve the psychometric properties of the data collection instrument authors gave a clear description of the constructs under investigation, attempted to standardize the data collection process and a sample that was representative of the study population.

## Conclusions

There was a high prevalence of WMSD among non-academic workers at the UZFMHS. Lower back pain and discomfort in the wrist and hands were the most reported problems. There was a significant association between reported pain and discomfort in specific body regions and sociodemographic factors such as age, gender, work experience, educational level and worker category. There were significantly increased odds of experiencing WMSDs among females, security personnel, catering staff and those with Bachleor’s degree, HND and ND qualifications. Increased work experience decreased the odds of experiencing WMSDs. Experiencing WMSDs was also influenced by gendered activities, cultural nuances and specific job demands of the various worker categories.

Our findings provide a clearer understanding of the relationship between the prevalence of WMSDs and sociodemographic factors. The high prevalence of WMSDs among non-academic workers points to the need for rehabilitation professionals within university health services. Occupational therapists and physiotherapists can design more focused ameliorative and health promotive interventions targeting at-risk populations in universities. Using their knowledge and techniques in musculoskeletal rehabilitation, occupational therapists and physiotherapists can reduce the disabling effects of WMSDs, such as absenteeism and frequent sick leave, thereby increasing efficiency and productivity among workers in the university and ultimately contributing to high-quality education. Occupational therapists should also suggest, after task and job analysis, redesigning work tasks and environments to reduce risk in populations highly likely to experience WMSDs. Examples include task shifting and microbreaks for security personnel or machine-assisted work and automated lifting for catering staff.

It is recommended that workers be more conscientious during the execution of their work tasks and avoid work postures and task sequences that increase their risk of developing WMSDs. Furthermore, employers should consider implementing wellness programs that aim to improve workers’ general physical health and reduce risks. Considerations should also be given to modifying both the work environment and routines to reduce the risk of WMSDs and increase health and well-being.

Future studies should also measure BMI and analyse its association with the prevalence of WMSDs in workers. The study should also be extended to all other worker categories in other work contexts that have not been included in this study. This will give occupational therapists and physiotherapists a better understanding of WMSDs, contributing to their professional reasoning in work rehabilitation and ergonomic interventions for workers of various categories.

### Supplementary Information

**Additional file 1.** Questionnaire on work related musculoskeletal symptoms and associated risk factors.

## Data Availability

The datasets used and analysed during the current study are available from the corresponding author upon reasonable request.

## Abbreviations

MSDs: Musculoskeletal disorders
WMSDs: Work-related musculoskeletal disorders
UZFMHS: University of Zimbabwe Faculty of Medicine and Health Sciences
SPSS: Statistical package for social sciences
NMQ: Nordic musculoskeletal questionnaire
JREC: Joint Parirenyatwa and Faculty of Medicine and Health Sciences Research Ethics Committee
GCE O`Level: General certificate of education ordinary level
HND: Higher national diploma
ND: National diploma
GCE A`Level: General Certificate of Education Advanced level
NC: National certificate
COVID-19: 2019 Coronavirus
BMI: Body mass index
