# Supplementary material for: Prevalence of work-related musculoskeletal disorders and associated factors among University of Zimbabwe Faculty of Medicine and Health Sciences non-academic workers: a cross-sectional study
Source: BMC Musculoskelet Disord. 2023 Oct 6;24:792. doi: 10.1186/s12891-023-06900-1 (PMC10557303; doi:10.1186/s12891-023-06900-1)
Supplement: Supplementary file 1 — Additional file 1. Questionnaire on work related musculoskeletal symptoms and associated risk factors. [file 12891_2023_6900_MOESM1_ESM.docx]

**DEPARTMENT OF PRIMARY HEALTH CARE SCIENCES** P O Box A178

Avondale

**REHABILITAION SCIENCES UNIT** Harare, Zimbabwe

Telephone:263 (242)791631


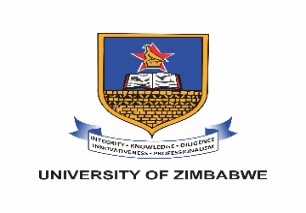
Ext. 2175/6

Email: [rehab@medsch.uz.ac.zw](mailto:rehab@medsch.uz.ac.zw)

**FACULTY OF MEDICINE AND HEALTH SCIENCES**

***TITLE* – QUESTIONNAIRE ON WORK RELATED MUSCULOSKELETAL SYMPTOMS AND ASSOCIATED RISK FACTORS**

**Date: ../……/…… QUESTIONNAIRE number:……..**

***You have been selected to participate in a study on work-related musculoskeletal disorders (WMSD) among workers at the institution. The purpose is to determine the prevalence of the WMSD and also determine the risk factors that might be associated with the development of these disorders. Please answer this questionnaire without consulting your colleagues. The questionnaire will only take you atleast 15 minutes to complete. Your cooperation is greatly appreciated***.

**SECTION A- Socio-Demographic characteristics of participants**

**Please fill in the spaces provides and tick where appropriate**

1.**Age** ………………years

**2.Gender:** Male Female

**3.Work experience in the job** ……………………years

**4.Highest Qualification attained**

PhD Masters Degree Higher National Diploma Diploma National Certificate A Level O Level

Junior Certificate Primary Education Certificate None

**5. Worker classification**:

Security

Catering

Driver

Library

Office worker

Technician

**SECTION B- Work related musculoskeletal symptoms**

1. **Have you at any time during the last 12 months had any pain/ discomfort/ ache in any of the following body parts? Please tick all that apply.**

| **Body part** | **RESPONSE** |  |
| --- | --- | --- |
|  | **NO** | **YES** |
| Neck |  |  |
| Shoulders |  |  |
| Wrists/ hands |  |  |
| Elbows |  |  |
| Lower back |  |  |
| Upper back |  |  |
| Thighs/ hips |  |  |
| Knees |  |  |
| Ankles |  |  |

1. Please tick on the appropriate box if you have experienced any **pain** on the listed **body parts** during the **past 3-months**

| **Body part** | **RESPONSE** |  |
| --- | --- | --- |
|  | **NO** | **YES** |
| Neck |  |  |
| Shoulders |  |  |
| Wrists/ hands |  |  |
| Elbows |  |  |
| Lower back |  |  |
| Upper back |  |  |
| Thighs/ hips |  |  |
| Knees |  |  |
| Ankles |  |  |

**SECTION C- Work duties and associated risk factors**

**1.This question is related to your working hours**.

a. How many days do you work in a week? ………………………….

b. Do you work in shifts? Yes No

If **yes**, how many shifts do you have week? ………………………….

c. How many **hours** do you work on a normal working **day**? …………hours

1. Do you have a scheduled work break for either tea or lunch during work?

Yes No

If yes, how long is it? ……………….. minutes

1. Do you at times take work breaks?

Yes No

**2. Below is a list of possible job duties you might perform. Please tick whether or not you perform the named task.**

| **Activity** | **Yes** | **No** |
| --- | --- | --- |
| Do you in your work have to lift/ carry heavy loads (5kgs) |  |  |
| Do you often have to work with your trunk in a bent posture for long period? |  |  |
| Do you often have to work with your neck in a bent position for long periods? |  |  |
| Do you often have to work standing in the same position for long periods of time? |  |  |
| Do you often have to work holding your wrist in the same position for long periods? |  |  |
| Do you often have to work while repeating the same movements with your arms and hands many times over a period of time? |  |  |
| Do you often have to work while sitting in the same position for a long period of time? |  |  |
| Do you often have to climb stairs several times a day at work? |  |  |
| Do you often have insufficient space to work on properly? |  |  |
| Do you often have to work with your neck in the same position for a long period of time? |  |  |
